# Effectiveness of intra-articular injections of sodium hyaluronate-chondroitin sulfate in knee osteoarthritis: a multicenter prospective study

**DOI:** 10.1007/s10195-015-0388-1

**Published:** 2015-11-14

**Authors:** Fabrizio Rivera, Luca Bertignone, Giancarlo Grandi, Roberto Camisassa, Guido Comaschi, Diego Trentini, Marco Zanone, Giuseppe Teppex, Gabriele Vasario, Giorgio Fortina

**Affiliations:** Department of Orthopedic Trauma, SS Annunziata Hospital, Savigliano, CN Italy; Sant’Anna Clinic, Casale Monferrato, AL Italy; Eporediese Hospital, Ivrea, TO Italy; La Vialarda Clinic, Biella, Italy; Sestri Ponente Hospital, Genova, Italy; Department of Orthopedics and Traumatology, IRCCS A.O.U. San Martino-IST, Genova, Italy; Department of Orthopedics and Traumatology, AO CTO Hospital, Turin, Italy; Santa Rita Clinic, Vercelli, Italy; Via Servais 200 A 16, Turin, Italy

**Keywords:** Knee osteoarthritis, Intra-articular injection, Sodium hyaluronate, Chondroitin sulfate

## Abstract

**Background:**

Intra-articular injection of hyaluronic acid is a well-established therapy for the treatment of knee osteoarthritis. The aim of the study was to assess the effectiveness and safety of the use of Arthrum HCS^®^ (40 mg hyaluronic acid and 40 mg chondroitin sulfate in 2 mL).

**Materials and methods:**

This was an open, multicenter, prospective study. Men or women over 40 years of age with documented knee osteoarthritis and WOMAC subscore A (severity of pain) ≥25 were enrolled. They received three weekly intra-articular injections of sodium hyaluronate 2 % and chondroitin sulfate 2 % in combination. WOMAC subscore A was assessed at 1, 3 and 6 months after the last injection.

**Results:**

One hundred and twelve patients were included (women, 66 %). The mean (SD) WOMAC subscore A decreased from 52.1 (15.2) at inclusion to 20.5 (19.7) at month 6 (*P* < 0.0001). The mean subscore was already significantly decreased 1 month after the last injection at 25.7 (*P* < 0.0001). Pain relief and consumption of analgesic drugs, both assessed with visual analogic scale (VAS), consistently decreased. The investigators were satisfied/very satisfied as regards the therapeutic effectiveness of sodium hyaluronate-chondroitin sulfate in reducing pain (77 %), improving mobility (78 %) and reducing the consumption of analgesics (74 %). Only one adverse effect was reported by one patient (knee tumefaction).

**Conclusion:**

These results suggest that intra-articular injections of Arthrum HCS^®^ (sodium hyaluronate plus chondroitin sulfate) in patients with knee osteoarthritis are efficient and safe. These results should be confirmed in a randomized controlled study.

**Level of evidence:**

IV.

## Introduction

It is estimated that around 250 million people in the world are affected by knee osteoarthritis [1]. Knee pain has an important impact by limiting activity and impairing quality of life. Thus, knee osteoarthritis has been identified as one of the medical conditions (with stroke, depression, hip fracture and heart disease) accounting for more physical disability than other diseases in people 65 years of age or older [2]. Moreover, knee osteoarthritis has a major impact on healthcare costs [3–6].

The management of osteoarthritis should be hierarchical, with non pharmacological methods as first interventions (weight loss, exercise and braces), followed by analgesic drugs [nonsteroidal anti-inflammatory drugs (NSAIDs) and other analgesics], local therapies (topical NSAIDs, intra-articular corticosteroids and hyaluronic acid), and surgery as a last resort [7–10].

Administration of exogenous hyaluronic acid (viscosupplementation) directly into the joint is available as a treatment for the symptoms of knee osteoarthritis. The purpose of viscosupplementation is to overcome the qualitative and quantitative deficiency of hyaluronic acid that is associated with osteoarthritis. Hyaluronic acid is a polysaccharide that is the main constituent of cartilage and synovial fluid; it is responsible for the mechanical properties of the joint by allowing shock absorption, lubrication and cartilage protection [11]. In osteoarthritis patients, synovial hyaluronate is depolymerized and is cleared at higher rates compared to normal subjects due to inflammation [12]. Intra-articular injections of hyaluronic acid have been shown to be as effective as NSAIDs with fewer systemic adverse events [13]; this therapy has a delayed onset of action in comparison with intra-articular corticosteroids, but a longer-lasting benefit [14]. Younger patients and patients at an earlier stage of the disease are more likely to benefit from viscosupplementation [15].

Arthrum HCS^®^ (LCA Pharmaceutical, Chartres, France) is a new specialty for viscosupplementation combining sodium hyaluronate and chondroitin sulfate. Chondroitin sulfate—a sulfated glycosaminoglycan—is an important structural component of the extracellular cartilage matrix.

On the articular system, chondroitin sulfate links to monomers with high molecular weights. The proteoglycan aggregate exhibits viscoelastic and hydration properties and an ability to interact with the surrounding tissue through electric charges, leading to protection of the cartilaginous tissues. Furthermore, chondroitin sulfates are inhibitors of extracellular proteases involved in the metabolism of connective tissues and stimulate proteoglycan production by chondrocytes in vitro; they also inhibit cartilage cytokine production and induce apoptosis of articular chondrocytes [16]. Preliminary clinical trials were in favor of the effectiveness of intra-articular injections of sodium hyaluronate-chondroitin sulfate. Thus, in a 3-month multicentric pilot study, a series of three weekly injections of a combination of hyaluronic acid-chondroitin sulfate was well tolerated and decreased pain in patients with knee osteoarthritis [17]. A recent clinical study suggested that a single injection of sodium hyaluronate-chondroitin sulfate in patients with lateral epicondylitis offer better pain benefits for 6 months after injection than intra-articular corticosteroids [18]. In an exploratory study, the effectiveness of intra-articular injections of a solution combining hyaluronic acid and chondroitin sulfate was assessed in 40 patients with knee osteoarthritis [19]. The clinical improvement, together with the changes of the ultrasound parameters and biomarkers of cartilage metabolism and joint inflammation, suggested a non-placebo effect. These results prompted us to assess in a prospective multicenter study the effectiveness and safety of the use of hyaluronic acid when combined with chondroitin sulfate in patients with knee osteoarthritis.

## Materials and methods

### Study design

This was an open, multicenter, prospective study, assessing the effectiveness of three intra-articular injections of sodium hyaluronate plus chondroitin sulfate (40 mg of each compound in 2 mL) in the symptomatic treatment of knee osteoarthritis.

The study was conducted prospectively by office or hospital specialists (orthopedic surgeons, rehabilitation medicine physicians) from October 2012 to December 2013.

Written informed consent was obtained from each patient. The protocol was conducted in accordance with the* Declaration of Helsinki and Guidelines on Good Clinical Practice* and approved by a local ethics committee.

### Inclusion criteria

Men or women over 40 years of age were eligible to participate if they: (1) had documented knee osteoarthritis evidenced with X-rays over the past 6 months with Kellgren-Lawrence score grade II or III [20]; (2) had pain and functional impairment for at least 3 months and Western Ontario and McMaster Universities Osteoarthritis Index (WOMAC) [21] subscore A (severity of pain) ≥25 (on a scale of 100); and (3) needed hyaluronic acid injections after the failure or intolerance to first-line analgesics or non steroidal anti-inflammatory drugs. The main exclusion criteria were: severe hydrarthrosis; inflammatory rheumatism; history of knee trauma in the past 6 months; history of arthroplasty or major surgery on the target knee in the past 6 months; history of arthroscopy or surgery on the target knee in the past 3 months; planned knee surgery during the study; history of septic arthritis of the knee; knee wound or skin condition; crural or sciatic radiculalgia of the lower limb; tendinopathy; symptomatic homolateral or contralateral hip disease; venous or lymphatic stenosis of the lower limb; medical history of venous thromboembolism (including pulmonary embolism) or patient with high risk of venous thromboembolism; patient with a history of auto-immune disease; treatment with diacerein, avocado soy unsaponifiables, glucosamine sulfate/chondroitin starting less than 3 months previously or with dosage modified during the past 3 months; recurrent episodes of chondrocalcinosis; previous treatment with viscosupplementation; injection of corticosteroids into the knee under study less than 3 months previously; known hypersensitivity to hyaluronic acid or substances with similar activity; ongoing anticoagulant therapy; pregnant or breastfeeding women.

### Treatment and clinical assessments

Demographic, description and history of knee osteoarthritis, concomitant treatments and WOMAC subscore A were recorded at inclusion visit. The WOMAC (Western Ontario and McMaster Universities) index is used to assess patients with osteoarthritis of the hip or knee [21]. The subscore A of the index is for pain assessment in five different circumstances: during walking (A1), using stairs (A2), in bed (A3), sitting or lying (A4) and standing (A5). For each item, pain is graded from 0 (none) to 100 (extreme). The sum for the five items is divided by five to give WOMAC subscore A.

Patients received three intra-articular injections of Arthrum HCS^®^ (40 mg hyaluronic acid and 40 mg chondroitin sulfate in 2 mL) 1 week apart. Assessment of treatment effectiveness and safety was performed during follow-up visits at 1 month, 3 months and 6 months after the last intra-articular injection.

The effectiveness assessment during the follow-up visits included: WOMAC subscore A, relief of pain using a visual analogic scale (VAS) ranging from 0 (“maximum relief”, i.e., no pain) to 100 (“no relief”, i.e., maximal pain) and consumption of analgesic drugs using a VAS ranging from 0 (“no consumption of analgesics”) to 100 (“maximal consumption of analgesics)”.

Adverse events were recorded immediately after the injections and during the follow-up visits.

### Statistical analysis

Data from previous studies were used to estimate the sample size [22, 23] With a loss to follow-up equal to 10 %, it was estimated that a sample size of 122 patients would provide 50 % power to detect a significant change of WOMAC subscore A (with alpha-risk at 5 %).

The primary endpoint was the change of WOMAC subscore A from inclusion to end of study. The secondary endpoints were the change of WOMAC subscore A from inclusion to month 1 or month 3, relief of pain at months 1, 3 and 6, consumption of analgesic drugs from baseline to months 1, 3 and 6 and global assessment by the investigator at the end of the study for the three criteria: pain reduction, improved mobility and consumption of analgesics.

Comparisons were made using Student’s *t* test for quantitative criteria and Chi^2^ test for non-ordinal qualitative variables (or Fisher’s exact test) and Wilcoxon’s test for ordinal data. The threshold for significance was set at 5 %.

The analyses were performed using SAS version 9.2 (SAS Institute, Cary, NC).

## Results

### Patient characteristics

Among 132 screened patients, 112 were analyzed (20 patients were <40 years of age and/or had a WOMAC subscore A <25).

The characteristics of patients at inclusion are summarized in Table 1. Two out of three patients were women and the mean age was 65.4 years (range from 44 to 88 years). Two-thirds of patients had a body mass index (BMI) above 25 kg/m^2^. The most frequent locations of knee osteoarthritis were the medial compartment (34.8 %; 39/112), tricompartmental (32.2 %; 35/112), and patello-femoral/medial femoro-tibial (10.7 %; 12/112). Radiological evaluation of osteoarthritis showed a Kellgren–Lawrence stage 2 in 64 (57, 1 %) cases, and a Kellgren–Lawrence stage 3 in 48 (42, 9 %) cases.

**Table 1:** Characteristics of patients at inclusion

Characteristic	*N* = 112
Age, years	
Mean (SD)	65.4 (10.6)
Median (range)	66 (44–88)
Female gender, *n* (%)	74 (66.1)
Body mass index^a^ (kg/m^2^)	
Mean (SD)	26.4 (4.1)
<18.5	3 (2.8)
(18.5–25)	32 (29.4)
(25–30)	57 (52.3)
≥30	17 (15.6)
Study knee, *n* (%)	
Right	67 (59.8 %)
Left	44 (3.3 %)
Right and left	1 (0.9 %)
Duration of knee osteoarthritis^b^, years, mean (SD)	3.0 (3.5)
Radiological stage, *n* (%)	
Grade II	64 (57.1 %)
Grade III	48 (42.9 %)
Prior knee surgery^b^ *n* (%)	29 (27.9 %)
Prior physical medicine and rehabilitation^c^	48 (43.6 %)
At least one analgesic drug within 3 months	87 (77.7 %)

^a^Missing data for three patients

^b^Missing data for eight patients

^c^Missing data for two patients

The mean duration of knee osteoarthritis was 3 years; 27.9 % (29/104) of patients underwent knee surgery (the main operation was meniscectomy; 35.7 %, 10/28). The median time between operation and inclusion was 7 years (*n* = 28). Just under half of patients had benefited from physical treatment and rehabilitation medicine (43.6 %; 48/110).

About four out of five patients (77.7 %; 87/112) were taking at least one analgesic treatment during the 3 months prior to the intra-articular injections: NSAIDs for 74.1 % (83/112) of patients and an analgesic other than NSAIDs for 34.9 % (38/109) of patients. There was a moderate relief due to the analgesic treatment: on a VAS from 0 (no relief) to 100 (maximal relief), the mean relief due to the analgesic treatment was 50.8 and 53.9 according to the investigator and the patient (*n* = 87), respectively.

### Severity of pain—WOMAC subscore A

On inclusion, the mean WOMAC subscore A was 52.1 (range 26–86). At 6 months, the mean WOMAC subscore A was 20.5 (range 0–80). Thus, the decrease of the subscore was −31.4 (*P* < 0.0001; Wilcoxon signed rank test).

**Fig. 1:**
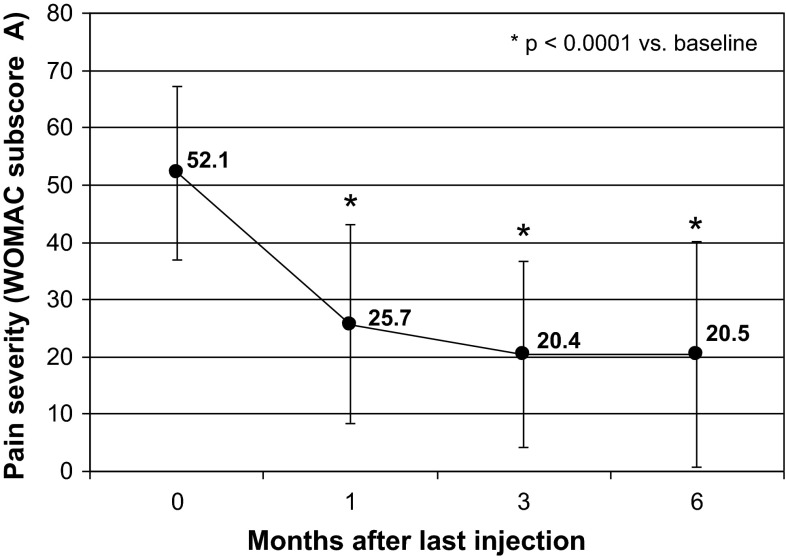
Pain severity (WOMAC subscore A) after three injections of sodium hyaluronate-chondroitin sulfate in knee osteoarthritis

The change in the WOMAC subscore A during the study is summarized in Fig. 1, and the changes in the five items of WOMAC subscore A (A1, walking; A2, using stairs; A3, in bed; A4, sitting or lying; A5, standing) are detailed in Table 2. One month after the last injection, the mean score decreased to 25.7 and pain continued to decrease with a mean score of 20.4 at 3 months. This decrease in the WOMAC subscore A at 1 month and 3 months was statistically significant compared to baseline (Wilcoxon signed rank test, *P* < 0.0001).

**Table 2:** Pain severity (WOMAC subscore A) after three intra-articular injections of sodium hyaluronate-chondroitin sulfate. Results are given as mean (SD). For each item, pain is graded from 0 (none) to 100 (extreme)

Time (months)	0	1	3	6
*N*	112	111	111	109
Items of WOMAC A				
A1 (walking)	46.0 (22.2)	23.3 (19.8)	17.7 (18.3)	16.7 (20.3)
A2 (using stairs)	72.1 (17.3)	37.0 (22.9)	31.0 (22.8)	31.0 (26.7)
A3 (in bed)	34.9 (25.2)	15.5 (19.0)	10.9 (17.7)	10.6 (18.0)
A4 (sitting or lying)	60.6 (21.4)	30.1 (21.7)	24.6 (19.4)	25.4 (23.8)
A5 (standing)	46.8 (23.5)	22.4 (22.5)	17.7 (18.8)	18.8 (22.2)
WOMAC A	52.1 (15.2)^a^	25.7 (17.4)^a^	20.4 (16.3)^a^	20.5 (19.7)^a^

^a^No treatment baseline score

### Pain relief

One month after the last intra-articular injection, the mean pain relief was assessed at 35.9 (25.6) on VAS by patients (0, maximal pain relief; 100, no relief). Pain relief continued to decrease at 3 and 6 months: 28.1 (23.1) and 26.1 (25.4), respectively. Compared to the values at 1 month, the values of pain relief at 3 and 6 months were statistically significant (*P* < 0.0001 and *P* = 0.0048, respectively).

### Consumption of analgesic drugs

One month after the last intra-articular injection, the patients assessed on a VAS their mean consumption of analgesic drugs from 0 (no consumption of analgesics) to 100 (maximal consumption of analgesics). The mean (SD) consumption decreased with time: 28.6 (24.4) at 1 month, 19.9 (21.7) at 3 months and 17.1 (22.3) at 6 months. Compared to the values at 1 month, the scores of the consumption of analgesic drugs at 3 and 6 months were significantly decreased (*P* < 0.0001 for both times).

### Global assessment by investigators

**Fig. 2:**
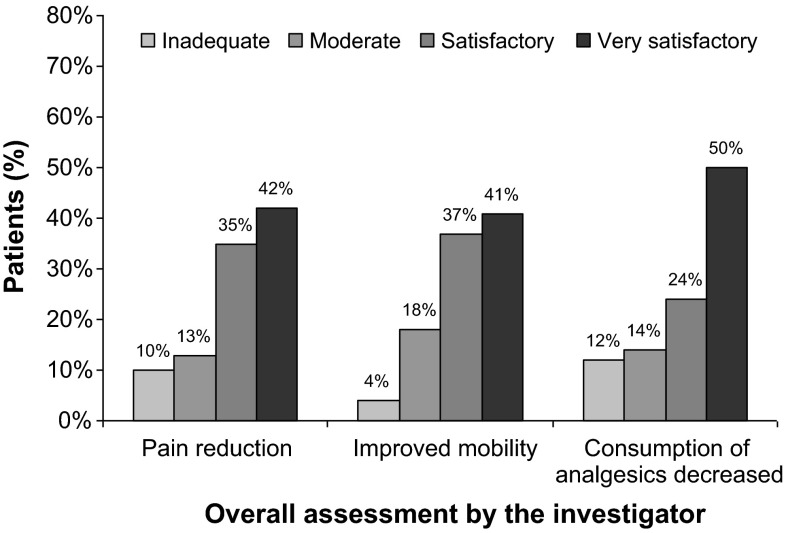
Overall assessment of efficacy of three injections of sodium hyaluronate-chondroitin sulfate in knee osteoarthritis by the investigator

The investigators were satisfied or very satisfied as regards the therapeutic effectiveness of sodium hyaluronate-chondroitin sulfate in reducing pain (77 %), improving mobility (78 %) and reducing the consumption of analgesics (74 %) (Fig. 2).

Overall, about 80 % of investigators stated that the results of the intra-articular injections of sodium hyaluronate-chondroitin sulfate combination were satisfactory or very satisfactory.

### Complications

One adverse effect was reported by one patient. This adverse event was knee tumefaction, which lasted 3 days after the first intra-articular injection.

## Discussion

Viscosupplementation with hyaluronic acid alone has demonstrated moderate but significant effectiveness vs placebo in terms of pain and function in knee osteoarthritis [11]. Our study is the first, to our knowledge, to assess the effectiveness and safety of injections of Arthrum HCS^®^ in a relatively large population of patients with knee osteoarthritis. We observed that the severity of pain assessed with the WOMAC subscore A decreased significantly from 52.1 to 20.5 at 6 months. The relief was already significant 1 month after the last injection. These results were confirmed by the assessment of pain relief and the decrease in the consumption of analgesics with VAS. Approximately three out of four investigators were satisfied/very satisfied as regards to the therapeutic effectiveness of the injections of sodium hyaluronate-chondroitin sulfate in reducing pain, improving mobility and reducing the consumption of analgesics.

The pilot study of Maheu et al. [17] in 41 patients with femoro-tibial knee osteoarthritis also reported an improvement 3 months after three 2-mL injections of hyaluronic acid (12 mg/mL) plus chondroitin sulfate (30 mg/mL). The mean VAS score decreased from 61 at baseline to 29 after 3 months (60 % of patients reported an improvement above 50 %). Although the dosages of the compounds were slightly different, these results are consistent with those of the present study.

The very low proportion of patients with adverse events confirms the safety of viscosupplementation in knee osteoarthritis. The harmlessness of viscosupplementation in knee osteoarthritis has been confirmed in a recent systematic review and meta-analysis of randomized saline-controlled trials for US-approved intra-articular hyaluronic acid [24]. There were no statistically significant differences between hyaluronic acid and saline controls for any safety outcome.

It is now debated that surgical procedures in knee osteoarthritis should be avoided as far as possible or at least delayed [25, 26]. The restoration of the viscoelasticity of the synovial fluid in order to protect cartilage, if possible during the early states of the disease, is an attractive therapeutic option. Moreover, a medico-economic evaluation showed that, together with clinical benefits, costs of knee osteoarthritis decreased after hyaluronic acid injections due to the decreased need for other treatments [23]. However no therapies have been shown to alter the natural history of osteoarthritis. In the absence of disease modifying osteoarthritis drugs, treatment of osteoarthritis is focused on controlling symptoms, especially pain [10, 27, 28]. Until prospective studies on the efficacy of hyaluronic acid on knee arthroplasty delay are completed, intra-articular treatment must be considered an additional non-operative strategy for relief of symptoms.

There are some limitations of the study. First, there was no control group. Indeed, there is a debate on the effectiveness of viscosupplementation in osteoarthritis, but some meta-analyses found an advantage of viscosupplementation over sham intervention [14, 24, 25]. Therefore, it was difficult to justify a sham control in one group. Nevertheless, the kinetics of the effect observed in the present study conform to the conclusions of a meta-analysis on viscosupplementation that showed that effectiveness became significant at 4 weeks, peaked at 8 weeks and persisted for 6 months [14]. Another limitation was the absence of demonstration of the benefit of the addition of chondroitin sulfate to hyaluronic acid. With a comparable total number of patients, the statistical power of the trial would decrease with an additional treatment group (hyaluronic acid alone). This issue should certainly be addressed in further studies.

Arthrum HCS^®^ is a new intra-articular treatment combining in the same injection two compounds that are deficient in osteoarthritis. Chondroitin sulfate is an essential component of cartilage and is present also in synovial fluid. Our results suggest that intra-articular injections of Arthrum HCS^®^ (sodium hyaluronate plus chondroitin sulfate) in patients with knee osteoarthritis allows a safe and effective control of pain. These results should be confirmed in a randomized controlled study.

## References

[1] Murray CJ, Vos T, Lozano R, Naghavi M, Flaxman AD, Michaud C (2012). Disability-adjusted life years (DALYs) for 291 diseases and injuries in 21 regions, 1990–2010: a systematic analysis for the Global Burden of Disease Study 2010. Lancet.

[2] Guccione AA, Felson DT, Anderson JJ, Anthony JM, Zhang Y, Wilson PW (1994). The effects of specific medical conditions on the functional limitations of elders in the Framingham study. Am J Public Health.

[3] Gupta S, Hawker GA, Laporte A, Croxford R, Coyte PC (2005). The economic burden of disabling hip and knee osteoarthritis (OA) from the perspective of individuals living with this condition. Rheumatology.

[4] Le Pen C, Reygrobellet C, Gerentes I (2005). Financial cost of osteoarthritis in France. The “COART” France study. Joint Bone Spine.

[5] Hunter DJ (2011). Osteoarthritis. Best Pract Res Clin Rheumatol.

[6] Hunter DJ (2015). Viscosupplementation for osteoarthritis of the knee. N Engl J Med.

[7] Bennell KL, Hunter DJ, Hinman RS (2012). Management of osteoarthritis of the knee. BMJ.

[8] Hochberg MC, Altman RD, April KT, Benkhalti M, Guyatt G, McGowan J (2012). American College of Rheumatology 2012 recommendations for the use of nonpharmacologic and pharmacologic therapies in osteoarthritis of the hand, hip, and knee. Arthritis Care Res (Hoboken).

[9] Zhang W, Nuki G, Moskowitz RW, Abramson S, Altman RD, Arden NK (2010). OARSI recommendations for the management of hip and knee osteoarthritis: part III: changes in evidence following systematic cumulative update of research published through January 2009. Osteoarthr Cartil.

[10] Jevsevar DS (2013). Treatment of osteoarthritis of the knee: evidence-based guideline, 2nd edition. J Am Acad Orthop Surg.

[11] Legre-Boyer V (2015). Viscosupplementation: techniques, indications, results. Orthop Traumatol Surg Res.

[12] Balazs EA, Denlinger JL (1993). Viscosupplementation: a new concept in the treatment of osteoarthritis. J Rheumatol Suppl.

[13] Pederzini LA, Milandri L, Tosi M, Prandini M, Nicoletta F (2013). Preliminary clinical experience with hyaluronan anti-adhesion gel in arthroscopic arthrolysis for posttraumatic elbow stiffness. J Orthopaed Traumatol.

[14] Bannuru RR, Natov NS, Obadan IE, Price LL, Schmid CH, McAlindon TE (2009). Therapeutic trajectory of hyaluronic acid versus corticosteroids in the treatment of knee osteoarthritis: a systematic review and meta-analysis. Arthritis Rheum.

[15] Wang CT, Lin J, Chang CJ, Lin YT, Hou SM (2004). Therapeutic effects of hyaluronic acid on osteoarthritis of the knee. A meta-analysis of randomized controlled trials. J Bone Joint Surg Am.

[16] Bali JP, Cousse H, Neuzil E (2001). Biochemical basis of the pharmacologic action of chondroitin sulfates on the osteoarticular system. Semin Arthritis Rheum.

[17] Maheu E, Zaïm M, Appelboom T, Bensaber M, Cadet C, Saurel A (2010) Evaluation of intra articular injections of hyaluronic acid and chondroitine sulfate for knee arthritis treatment: a multicentric pilot study with 3 monts follow-up. National rheumatolgy meeting 2010 (Société Française de Rhumatologie). http://www.rhumatologie.asso.fr/data/ModuleProgramme/PageSite/2010-1/Resume/6007.asp. Accessed* date* 2015

[18] Tosun HB, Gumustas S, Agir I, Uludag A, Serbest S, Pepele D (2015) Comparison of the effects of sodium hyaluronate-chondroitin sulphate and corticosteroid in the treatment of lateral epicondylitis: a prospective randomized trial. J Orthop Sci 20:837–843 10.1007/s00776-015-0747-z 26133944

[19] Henrotin Y, Hauzeur JP, Bruel P, Appelboom T (2012). Intra-articular use of a medical device composed of hyaluronic acid and chondroitin sulfate (Structovial CS): effects on clinical, ultrasonographic and biological parameters. BMC Res Notes.

[20] Ahlbäck S (1968). Osteoarthrosis of the knee: a radiographic investigation. Acta Radiol Stockholm.

[21] McConnell S, Kolopack P, Davis AM (2001). The Western Ontario and McMaster Universities Osteoarthritis Index (WOMAC): a review of its utility and measurement properties. Arthritis Rheum.

[22] Ehrich EW, Davies GM, Watson DJ, Bolognese JA, Seidenberg BC, Bellamy N (2000). Minimal perceptible clinical improvement with the Western Ontario and McMaster Universities osteoarthritis index questionnaire and global assessments in patients with osteoarthritis. J Rheumatol.

[23] Mazieres B, Bard H, Ligier M, Bru I, d’Orsay GG, Le Pen C (2007). Medicoeconomic evaluation of hyaluronic acid for knee osteoarthritis in everyday practice: the MESSAGE study. Joint Bone Spine.

[24] Miller LE, Block JE (2013). US-approved intra-articular hyaluronic acid injections are safe and effective in patients with knee osteoarthritis: systematic review and meta-analysis of randomized, saline-controlled trials. Clin Med Insights Arthritis Musculoskelet Disord.

[25] Lo GH, LaValley M, McAlindon T, Felson DT (2003). Intra-articular hyaluronic acid in treatment of knee osteoarthritis: a meta-analysis. JAMA.

[26] Abbott Thomas, Altman Roy D, Dimeff Robert, Fredericson Michael, Vad Vijay, Vitanzo Peter (2013). Do hyaluronic acid injections delay total knee replacement surgery? [abstract]. Arthritis Rheum.

[27] American College of Rheumatology Subcommittee on Osteoarthritis Guidelines (2000). Recommendations for the medical management of osteoarthritis of the hip and knee. Arthritis Rheum.

[28] Zhang W, Nuki G, Moskowitz RW, Abramson S, Altman RD, Arden NK (2010). OARSI recommendations for the management of hip and knee osteoarthritis: part III: changes in evidence following systematic cumulative update of research published through January 2009. Osteoarthr Cartil.

