# Erratum to: Effectiveness of intra-articular injections of sodium hyaluronate-chondroitin sulfate in knee osteoarthritis: a multicenter prospective study

**DOI:** 10.1007/s10195-015-0390-7

**Published:** 2016-01-11

**Authors:** Fabrizio Rivera, Luca Bertignone, Gianclaudio Grandi, Roberto Camisassa, Guido Comaschi, Roberto Trentini, Marco Zanone, Giuseppe Teppex, Gabriele Vasario, Giorgio Fortina

**Affiliations:** Department of Orthopedic Trauma, SS Annunziata Hospital, Savigliano, CN Italy; Sant’Anna Clinic, Casale Monferrato, AL Italy; Eporediese Hospital, Ivrea, TO Italy; La Vialarda Clinic, Biella, Italy; Sestri Ponente Hospital, Genova, Italy; Department of Orthopedics and Traumatology, IRCCS A.O.U. San Martino-IST, Genova, Italy; Department of Orthopedics and Traumatology, AO CTO Hospital, Turin, Italy; Santa Rita Clinic, Vercelli, Italy; Via Servais 200 A 16, Turin, Italy

## Erratum to: J Orthopaed Traumatol DOI 10.1007/s10195-015-0388-1

Unfortunately, the first names of two co-authors, Prof. Trentini and Prof. Grandi were incorrectly published in the original publication. The correct names of these two authors are given below.

Roberto Trentini and Gianclaudio Grandi

